# Highly Educated Men Establish Strong Emotional Links with Their Dogs: A Study with Monash Dog Owner Relationship Scale (MDORS) in Committed Spanish Dog Owners

**DOI:** 10.1371/journal.pone.0168748

**Published:** 2016-12-29

**Authors:** Paula Calvo, Jonathan Bowen, Antoni Bulbena, Adolf Tobeña, Jaume Fatjó

**Affiliations:** 1 Chair Affinity Foundation Animals and Health, Department of Psychiatry and Legal Medicine, Universitat Autònoma de Barcelona, Bellaterra, Catalunya, Spain; 2 IMIM (Hospital del Mar Medical Research Institute), Parc de Recerca Biomèdica de Barcelona, Barcelona, Catalunya, Spain; 3 Royal Veterinary College, Hawkshead Lane, North Mimms, Hertfordshire, United Kingdom; 4 INAD (Institut de Neuropsiquiatria i Addiccions), Parc de Salut Mar, Barcelona, Catalunya, Spain; University of Portsmouth, UNITED KINGDOM

## Abstract

The characteristics of the human-animal bond may be influenced by both owner-related and dog-related factors. A study was designed to explore the existence of different dog ownership patterns and their related factors. We created an on line questionnaire that included demographic questions about the dog and the owner, a Spanish version of the Monash Dog Owner Relationship Scale (MDORS) and a validated measure of satisfaction with life (Cantril’s ladder). We collected 1140 valid responses from adult dog owners, who were recruited using the client databases of Spanish veterinary practices. We explored the presence of groups within the population using Principal Components Analysis (PCA) of the MDORS variables combined with Hierarchical Cluster Analysis (HCA). Two groups were found; Group I having a higher level of emotional involvement with their dogs compared with Group II. Binary logistic regression was used to explore demographic factors that influenced group membership. Four variables were significantly associated with membership of Group I (p<0.0001); male gender of the owner (OR = 32.36), high school level of maximum educational attainment (OR = 0.052), university level of maximum educational attainment (OR = 8.652), and owner Cantril’s score (OR = 0.807). The results obtained from this convenience sample demonstrate that different patterns of dog-ownership may be present within a population of owner-dog dyads, and that certain owner characteristics are associated with the type of owner-dog relationship. Future research could apply a similar approach to different types of sample population in order to identify specific patterns of dog-ownership.

## Introduction

Dogs have become an important component of western societies and a large proportion of homes include a pet dog. According to recent estimates, 21% of European households [1] and 36.5% of US households own a dog [2]. With 31% of households owning a pet dog, Spanish levels of dog ownership are typical for Europe [3]. Since differences in the patterns of relationships between dogs and owners have implications both for human and canine welfare and health, the formation, nature and consequences of human-dog relationships have been receiving increasing scientific attention [4]. Pet ownership has been shown to have potential benefits for human psychological and physical wellbeing [5] including a range of benefits for cardiovascular health [6]. However, whilst many human-dog relationships are successful, some fail and lead to relinquishment or abandonment [7]. It is therefore important to broaden our knowledge of patterns of dog ownership to understand those factors that may contribute to the success or the failure of human-dog relationships.

Emerson’s social exchange theory considers a relationship to be successful if there is a positive balance between the benefits and costs of that relationship [8]. If a relationship’s costs outweigh its rewards, then people tend to terminate or abandon it. The social exchange theory has been applied to pet-owner relationship, with researchers investigating the benefits and the costs of dog ownership and developing methods to assess the balance between benefit and cost for dog-owner dyads [4, 9].

Benefits of dog ownership include the dog’s unconditional display of affection and acceptance, non-judgmental love and demand for interaction. The social support provided, together with increased social interaction, lead to positive physiological, neurochemical and psychological effects [7, 10].

Costs of dog ownership include accommodation issues, impediments to lifestyle, changes in social/family network and/or financial factors [7, 11, 12]. The study of owner-dog relationship profiles offers an alternative method to study the costs and negative aspects of dog ownership, and thereby to bring to light important factors contributing to a successful owner-dog relationship.

It has been found that certain owner factors, such as age, sex, income/social class, marital status, rural/urban residence and household type, can have an effect on the profile of pet ownership [13–15]. The Monash Dog Owner Relationship Scale (MDORS) was created and validated for the assessment of human-companion dog relationships. This scale follows the principles of the social exchange theory and includes subscales relating to owner-dog interaction, emotional closeness and perceived costs [4].

Previous research on dog-ownership, using MDORS or other tools, has focused on different aspects of the human-animal bond, including: 1) analysis of the psychological characteristics of this bond [16], 2) relationship with hormone levels (cortisol and oxytocin) [16], 3) relationship with behaviour problems [17], 4) relationship with dog and owner characteristics [17, 18], 5) relationship with responsible ownership practices [19], and 6) relationship between owner and dog perspectives [20].

The aim of the present cross-sectional study was to look for different patterns of owner-dog relationship and to identify those owner and/or dog dependent factors, which influence the quality and type of relationship from the owner’s perspective. To make a simple assessment of the owner’s satisfaction with life, and to explore the relationship between this and characteristics of the dog-owner bond, we included Cantril’s Self Anchoring Ladder [21].

We found two patterns of dog-owner relationship that differed in quality, mainly of the emotional aspect of the relationship, as well as those owner and dog characteristics, which were associated with those patterns. These results highlight factors to take into account when predicting the development of the relationship between a dog and its owner and further research of this kind may identify risk factors for dog abandonment and relinquishment. Given that the methodology of this study produced interesting and insightful results with a convenience sample, future research could apply the same approach to different kinds of populations to explore other forms of human-dog interactions, such as between service users and their assistance dogs.

## Materials and Methods

### Ethics statement

Permission to perform this study was sought, and obtained, from the Research Ethics Committee of the Department of Psychiatry and Legal Medicine at the Universitat Autònoma de Barcelona and the Clinical Research Ethics Committee of the Hospital del Mar Medical Research Institute.

Survey participants were fully informed about the purpose and background of the study by e-mail. As the study survey was entirely anonymous, informed signed consent was not required from participants. Participants were also able to abandon the online survey anytime.

### Subjects

From April to November 2013, the Spanish Small Animals Veterinary Association (AVEPA) contacted its member veterinary practices to recruit them to take part in the study. Participating clinics were given instructions to e-mail their clients with a request to answer an anonymous online survey. During the client enrolment period, which was from April 2013 until January 2014, a total of 1850 adult current dog owners participated.

### Materials

The online survey consisted of four sections:

- Owner demographics and characteristics of pet-ownership, such as sex, age, maximum level of education, family role, regional location, type of residential area (e.g. city/town/rural), and duration of ownership.
- The characteristics of the dog, such as sex, neuter status, age, size and breed.
- A standardized, back-translated Spanish language version of MDORS questionnaire [4]. The MDORS is a 28-item five point Likert multidimensional scale to measure the owner’s perceived relationship with their dog. The MDORS includes three sub-scales related to separate dimensions of the human-dog relationship; Owner-dog Interaction, Perceived Emotional Closeness, and Perceived Costs. There is no existing data to determine level of relationship quality (e.g. high, medium or low) from MDORS scores. So, scores can only be compared within a specified group of human-dog dyads. Higher scores in any of the three sub-scales of the MDORS indicate a positive perception with respect to that subscale, even if those variables belong to the sub-scale of perceived costs of MDORS. Higher scores in the Interaction Level sub-scale of MDORS mean higher level of interaction, higher scores in the Perceived Emotional Closeness sub-scale of MDORS mean higher emotional closeness, and higher scores in the Perceived Costs sub-scale of MDORS mean lower perceived costs for the owner.
- A validated Spanish version of Cantril’s Self Anchoring Ladder [21, 22]. This psychometric tool consists of a single question to evaluate overall satisfaction with life. Respondents are asked to report their current level of satisfaction with life on a scale from zero (worst possible life) to 10 (best possible life). Satisfaction with life is considered one of the two major aspects of subjective wellbeing, with the other being emotional wellbeing [23].

### Data analysis

When the client enrolment period ended in January 2014, three inclusion criteria were applied to select a population that was suitable for the intended analysis; respondents had to be from Spain, they had to have owned the dog for at least one year (to ensure there was a stable dog-ownership pattern), and they had to be 25 years of age or older. A high minimum age had to be specified to ensure that respondents were old enough for their maximum level of educational attainment and family role to be meaningful, and not merely a reflection of the limitations imposed by their age at the time of responding. Responses with inconsistent or incomplete data were also eliminated (for example when duration of ownership exceeded the dog’s present age). This left a total of 1140 survey responses.

A total score and scores for each of the MDORS subscales were calculated according to the protocol set out by the authors of that scale [4].

**Fig 1:**
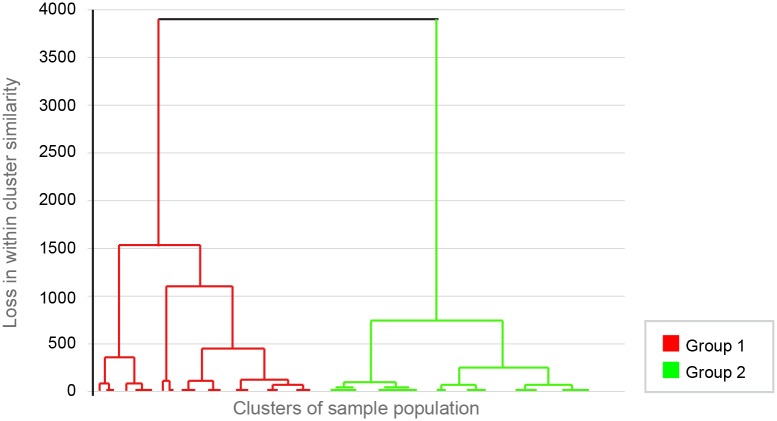
Dendrogram plot generated from the hierarchical cluster analysis of Principal Component analysis of MDORS results of the population study: Clusters distribution. This dendrogram represents the result of the HCA (Hierarchical cluster analysis). The plot shows the distribution of the sample population in different clusters. The sample population appears principally distributed in two groups (two big clusters) after PCA-HCA: Group I (higher emotional dog-owner bond) and Group II (lower emotional dog-owner bond). The vertical axis indicates the loss in within cluster similarity (i.e. the variance increase, when clusters are merged). The horizontal axis represents the cluster groups of all the individuals of the sample population.

Descriptive statistics were performed using Graphpad Prism 6. Prior to hierarchical clustering, all 28 items of the MDORS were included as variables in a Principal Components Analysis (PCA) model. This was for dimension reduction, to create a smaller number of variables that summarised the systematic relationships in the MDORS data. For the PCA, all data was first unit-variance scaled and mean centred. The model was automatically generated in SIMCA P+12, with the final number of principal components being determined by the point at which the addition of further principal components did not increase the cumulative value of Q^2^ for the total number of components (as a measure of goodness of prediction) A two component model was generated, with the “emotional closeness” sub-scale items providing the strongest loadings for the first principal component and the “perceived costs” sub-scale items primarily loading onto the second principal component. Scores for the two principal components were then used for HCA (Ward’s method, also performed within SIMCA P+12). A dendrogram plot (Fig 1) was generated, with the vertical axis set to indicate the loss in within-cluster similarity (the variance increase) as clusters merged. This was used to select the cluster solution for the later analysis; a two-cluster solution was chosen as this offered the greatest between cluster distance. The resulting model was explored and validated using Projection to Latent Structures-Discriminant Analysis (PLS-DA) to establish which MDORS variables contributed most to the difference between the two groups. Binary Logistic Regression (BLR. Enter method; SPSS 22) was used to identify those owner and dog characteristics which contributed to group membership.

## Results

### Recruitment process

We obtained 1850 completed questionnaires. After filtering all those responses using the pre-established inclusion criteria, we had 1140 completed and valid responses for analysis, which was considered the final study population.

### Demographics

There was a sex bias in the respondent population, with 28.30% men and 71.70% women. The mean age of respondents was: 39.86 (SD = 10.24). Demographic characteristics of the sample are shown in Table 1. Demographic data for the Spanish national population (citizens of 25 years old or more) for 2013 (year of the study data collection) have been included in Table 1 to allow comparison with the demographics of the study [24].

**Table 1:** Dog Owners’ Demographics of the study sample.

	N	% of the study population	% Spanish population (2013)
**SEX**			
Men	**324**	28.30	49.48
Women	**818**	71.70	51.43
**AGE** Mean 39.86 (SD = 10.24)			
25 to 40 years old	**649**	56.92	30.08
41 to 65 years old	**479**	42.01	31.26
> 65 years old	**12**	1.05	38.65
**FAMILY ROLE**			
Couple without children	**426**	37.36	21.64
Couple with children	**372**	32.63	34.92
Live alone	**136**	11.92	24.2
Son/daughter living with parents	**136**	11.92	-
Single-parent family	**75**	6.57	9.37
Share household with no relatives	**14**	1.22	3.09
Grandparent at family home	**11**	0.96	-
Other role	**28**	2.45	6.73
**MAXIMUM ATTAINED EDUCATION LEVEL**			
University	**658**	57.71	33.70
Vocational training	**224**	19.64	-
High school	**169**	14.82	21.70
Basic level	**89**	7.80	44.60
**TYPE OF AREA**			
Urban	**769**	67.45	-
Rural	**372**	32.63	-
**SPANISH REGION**			
Centre	**374**	38.80	23.54
North East	**326**	28.59	18.80
Eastern Coast	**161**	14.12	13.79
North	**110**	9.64	8.18
South	**90**	7.89	20.75
North West	**55**	4.82	8.18
Canary Islands	**15**	1.31	4.54
Balearic Islands	**9**	0.78	2.39

There was an almost equal split between male and female dogs, and pure and crossbred dogs; 41.31% were crossbreed dogs and 58.68% were pure breed dogs, the latter comprising 35 different breeds of which the most common was the Yorkshire Terrier (n = 79). Size of dogs was classified according to the dog’s weight. Characteristics of the dogs of the sample are shown in Table 2.

**Table 2:** Demographics of dogs of the study sample.

	Total number	Percentage
**SEX**		
Male	**554**	48.59%
Female	**586**	51.40%
**BREED**		
Cross breed	**471**	41.31%
Pure breed	**669**	58.68%
**SIZE**		
Mini (< 2kg)	**30**	2.63%
Small (2-10kg)	**415**	36.40%
Medium (11-25kg)	**372**	32.63%
Large (26-50kg)	**304**	26.66%
Giant (>50kg)	**19**	1.66%
**AGE** Mean 5.43 (SD = 3.62)		
1–5 years old	**679**	59.56%
6–10 years old	**314**	27.54%
>10 years old	**147**	12.89%
**TIME OF OWNERSHIP**		
1–2 years	**354**	31.05%
3–5 years	**381**	33.42%
6–10 years	**279**	24.47%
>10 years	**126**	11.05%

### Cantril’s self-anchoring Scale

**Fig 2:**
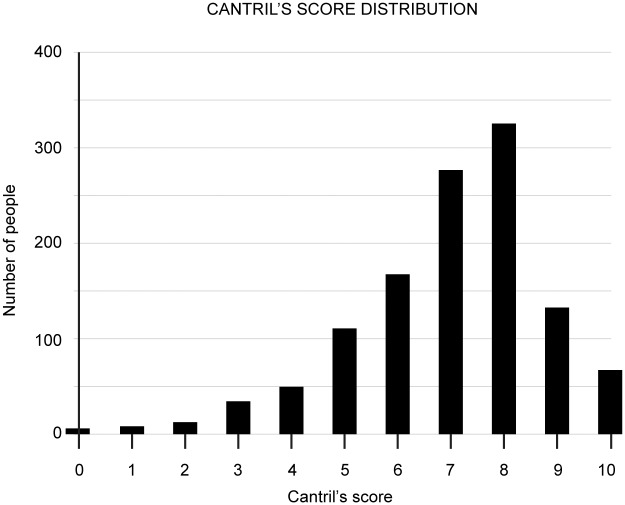
Cantril’s score (Satisfaction of life score) distribution of the dog-owners of the sample population.

Mean score for Cantril’s Self-Anchoring Scale was 7.03 (SD = 1.713). See Fig 2 for the distribution of values.

### Dog-owner relationship: MDORS results

Mean scores for single items, subscales, and the total MDORS score are summarized in Table 3.

**Table 3:** MDORS results: Mean scores for single items, subscales and the total MDORS score.

MDORS ITEM	Mean (SD)
**Sub-scale I: Dog-Owner Interaction** (Score range: 9–45)	**36.35 (4.76)**
How often do you kiss your dog?	**4.20 (1.50)**
How often do you play games with your dog?	**4.79 (0.61)**
How often do you take your dog to visit people?	**3.97 (1.36)**
How often do you buy your dog presents?	**3.16 (1.04)**
How often do you give your dog food treats?	**4.10 (1.19)**
How often do you take your dog in the car?	**3.25 (1.16)**
How often do you groom your dog?	**3.13 (1.18)**
How often do you hug your dog?	**4.83 (0.58)**
How often do you have your dog with you while relaxing, i.e. watching TV?	**4.88 (0.53)**
**Sub-scale II: Emotional Closeness** (Score range: 10–50)	**44.70 (5.27)**
My dog helps me get through tough times.	**4.54 (0.69)**
My dog is there whenever I need to be comforted.	**4.63 (0.66)**
If everyone else left me, my dog would still be there for me.	**4.74 (0.58)**
I would like to have my dog near me all the time.	**4.22 (0.97)**
My dog provides me with constant companionship.	**4.39 (1.21)**
How often do you tell your dog things you don’t tell anyone else?	**4.28 (1.34)**
My dog is constantly attentive to me.	**4.19 (0.94)**
How traumatic do you think it will be for you when your dog dies?	**4.67 (0.55)**
My dog gives me a reason to get up in the morning.	**4.36 (0.85)**
I wish my dog and I never had to be apart.	**4.64 (0.73)**
**Sub-scale III: Perceived Costs** (Score range: 9–45)	**38.18 (4.98)**
How often do you feel that looking after your dog is a chore?	**4.66 (0.80)**
It is annoying that I sometimes have to change my plans because of my dog.	**4.09 (0.95)**
How often does your dog stop you doing things you want to?	**4.41 (0.81)**
There are major aspects of owning a dog I don’t like.	**4.22 (0.94)**
It bothers me that my dog stops me doing things I enjoyed doing before I owned it.	**4.30 (0.87)**
My dog costs too much money.	**3.51 (1.13)**
My dog makes too much mess.	**4.08 (1.00)**
How often do you feel that having a dog is more trouble than it is worth?	**4.76 (0.66)**
How hard is to look after your dog?	**4.16 (0.78)**
**Total MDORS (out of a possible 140)**	**119.20 (11.07)**

The scoring system corresponds to the original MDORS scale [4]. Each item has a score range between 1 and 5. Score ranges for each sub-scale are included too. (SD = standard deviation)

All MDORS variables were included in a PCA, This produced a two component model (R^2^ = 0.419, Q^2^ = 0.198). See Table 4 for results of loadings of the PCA.

**Table 4:** Results of the PCA of MDORS variables results of the population of the study.

MDORS Item	PC 1	PC 2
**1. I wish my dog and I never had to be apart**	0.281048	-0.105922
**2. I would like to have my dog near me all the time**	0.280355	-0.133206
**3. My dog helps me get through tough times.**	0.275265	-0.093508
**4. My dog is there whenever I need to be comforted.**	0.267292	-0.144414
**5. How traumatic do you think it will be for you when your dog dies?**	0.254623	-0.124108
**6. My dog gives me a reason to get up in the morning**	0.254571	-0.100460
**7. If everyone else left me my dog would still be there for me**	0.241787	-0.120916
**8. How often do you hug your dog?**	0.215651	-0.113394
**9. How often do you tell your dog things you don't tell anyone else?**	0.207531	-0.165469
**10. There are major aspects of owning a dog I don't like**	0.199263	-0.187088
**11. How often do you buy your dog presents?**	0.186887	-0.130078
**12. My dog is constantly attentive to me.**	0.168597	-0.128240
**13. How often do you play games with your dog?**	0.154548	-0.045927
**14. How often do you have your dog with you while relaxing?**	0.151960	-0.054691
**15. How often do you take your dog to visit people?**	0.150283	-0.029251
**16. How often do you kiss your dog?**	0.141473	-0.133000
**17. How often do you groom your dog?**	0.129786	-0.018630
**18. How often do you give your dog food treats?**	0.119461	-0.093175
**19. My dog provides me with constant companionship.**	0.100983	-0.095220
**20. How often do you take your dog in the car?**	0.087641	-0.062846
**21. How often does your dog stop you doing things you want to?**	0.119011	0.378664
**22. It bothers me that my dog stops me doing things I enjoyed doing before I owned it**	0.171475	0.362851
**23. It is annoying that I sometimes have to change my plans because of my dog**	0.202842	0.348500
**24. It is annoying that I sometimes have to change my plans because of my dog**	0.172377	0.304502
**25. My dog makes too much mess.**	0.107552	0.293268
**26. How hard is it to look after your dog?**	0.157785	0.252871
**27. How often do you feel that having a dog is more trouble than it is worth?**	0.135970	0.239314
**28. My dog costs too much money**	0.080734	0.224907

The Table shows the loadings of each variable of the MDORS for each factor (PC1 and PC2) detected by the PCA. Main loadings for each PC are shaded yellow. (PC = Principal Component)

**Fig 3:**
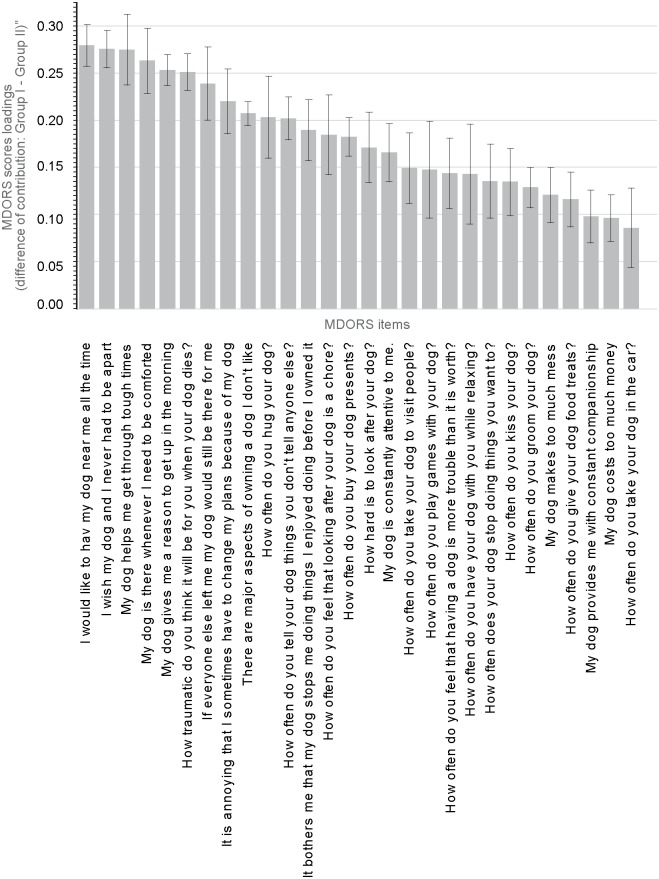
Difference in contribution of MDORS (Monash Dog Owner Relationship Scale) variables of Group I compared to Group II. This graph shows the difference in contribution (score loadings) of each MDORS variable in Group I (higher emotional dog-owner bond) in comparison to Group II (lower emotional dog-owner bond). Group I was associated with higher loadings for all 28 of the MDORS variables, but main differences were found in the loadings for the emotional aspects of MDORS.

Scores for the two principal components were then used in hierarchical clustering. A dendrogram (Fig 1) was used to visually select the solution with the greatest distance between clusters. This resulted in a model with two clusters (groups); Group I accounted for 56.1% of the population and Group II for 43.9% of the sample population (Fig 1). The validity of these groupings was tested using PLS-DA, which produced a model with two predictive components (R^2^Y = 0.611 Q^2^ = 0.592). The reliability of the PLS-DA model was confirmed using a permutations method, in which the values of R^2^Y and Q^2^ of models with randomly permutated class membership were compared with values for the real model. The real model outperformed all of the permutated models. As further confirmation of the quality of the model, analysis of variance of cross-validated residuals (CV-ANOVA) for the PLS-DA model was highly significant (p<1x10^-20^). To improve interpretability, an orthogonal signal correction filter was applied to create an O-PLS-DA model with a single predictive component (R^2^Y = 0.595, Q^2^ = 0.589, CV-ANOVA p<1x10^-20^). This showed that Group I was associated with higher loadings for all 28 of the MDORS variables. A bar chart of loadings from the O-PLS-DA model shows those items which contributed most strongly to the discrimination between Groups I and II (Fig 3), with items such as “I would like to have my dog near me all the time”, “My dog helps me get through tough times”, and “My dog is there whenever I need to be comforted” featuring most strongly.

A binary logistic regression (BLR) model was then used to identify owner and dog characteristics that were associated with membership of the two groups defined by PCA-HCA. Variables were selected for inclusion in the binary logistic regression model (Enter method) using appropriate univariate contrasts; only variables for which there was a significant difference between Groups I and Group II were included (p<0.1, as a standard approach for BLR). Classification accuracy for the model was 83.8%, Nagelkerke R^2^ was good (0.66), and the model passed an omnibus test (chi-square = 707.44, df = 8, p<0.0001). According to the model, four variables, presented here in descending order of influence, were found to contribute significantly to membership of Group I; university level of maximum educational attainment (OR = 8.65, p<0.0001), male owner (OR = 32.36, p<0.0001), high school level of maximum educational attainment (OR = 0.05, p<0.0001), and owner Cantril’s score (OR = 0.81, p<0.0001) Table 5. According to the model, the rest of the variables included in the demographic questionnaire, such as dog characteristics (e.g. age, sex, duration of ownership, owner’s age or owner’s family role, did not contribute to membership to any of the two identified distinct groups (Group I and Group II).

**Table 5:** Results of binary logistic regression analysis.

Variables	S.E.	Significance (p)	OR
Owner sex: male	0.331	<0.0001	32.359
Family role: One parent family	0.347	0.5710	0.822
Family role: flat mate	0.805	0.3260	0.454
Maximum education level: basic	3737.680	0.9950	0.000
Maximum education level: high school	0.405	<0.0001	0.052
Maximum education level: University	0.207	<0.0001	8.652
Dog size: mini	0.588	0.0710	2.892
Cantril’s score (1 point increase)	0.056	<0.0001	0.807

This table shows the probability of each variable to be present in a dog-owner of Group I (more emotionally dependent dog-owner relationship).

S.E. = Standard error; OR = Odds Ratio

## Discussion

The main aims of this research were to find groups within the population with respect to the perceived relationship with their dog, assessed as a balance of costs and benefits of the relationship (social exchange theory) [8], and to identify dog and owner factors that might be associated with each pattern.

As the main result of our study, we found two patterns of dog-owner relationship that differed in quality, mainly of the emotional aspect of the relationship, as well as those owner and dog characteristics, which were associated with those patterns. These results highlight factors to take into account when predicting the development of the relationship between a dog and its owner and further research of this kind may identify risk factors for dog abandonment and relinquishment. Given that the methodology of this study produced interesting and insightful results with a convenience sample, future research could apply the same approach to different kinds of populations to explore other forms of human-dog interactions, such as between service users and their assistance dogs.

### Demographics of the population of the study

Since this study was conducted with a convenience sample, it is interesting to compare our sample population with Spanish population official statistics for the year 2013 (the year that this study was conducted), to gain an impression of biases present in our convenience sample. As shown in Table 1, some characteristics of our convenience sample are overrepresented in comparison to the census characteristics of the 2013 Spanish population; female gender, university education level, people from North East and Central Spain, couples living without children and people living alone.

Although women were overrepresented (71.7% of respondents), the proportion of male and female owners was similar to previous studies of pet ownership in which recruitment was voluntary [4, 25–29]. Evidence suggest that women may, in general, be more willing to participate in online surveys than men [30]. In addition, given that recruitment was through veterinary clinics, perhaps the recruitment favoured women because they were more involved in their pet’s care and visits to the clinic. Given that mothers may be more involved and engaged with childcare than fathers [31] and the relationship between people and their dogs includes a pattern of care-giving that is similar to that between parents and children [32–34], perhaps women are more involved in dog care, because they tend to be more involved in the childcare tasks. Another possible reason for the overrepresentation of women in this study could be sex differences in the use of social media and internet resources. Previous research has suggested that women use internet tools more for private social connectivity than men [35, 36]. As pet ownership is part of the spectrum of a person’s private social life, the use of internet-based methods to recruit cases could not only capture the interest of women more than men, but also favour the dissemination of information through female social networks.

A majority of the study participants were university educated (57.71%). This overrepresentation of highly educated people agrees with previous research; for example, in a study of a self-selected sample of 1016 adult dog owners in Australia, 62% of the participants had a university level of education [19]. This could indicate that dog ownership engagement and interest is greater in people with higher levels of education, but it may simply relate to differences in the use of internet resources by people with different educational backgrounds and occupational status [35, 36]. Some studies have shown that more educated and more affluent people are more likely to participate in surveys [30]. With pet-ownership being costly, there may also be an effect of differential exposure to dogs during childhood; families with higher socio-economic status may be more able to afford to own a dog and provide their children with the resources needed to achieve higher education levels. Some research supports the link between socioeconomic status and pet ownership [15], although not all previous research has shown this direct correlation between socio-economic status and dog owning [15, 37]. This point will be discussed later in this section, when considering the factors related to quality of owner-dog relationship.

Being part of a couple without children or living alone were family roles that were over-represented in our study, compared with national Spanish demographic data. This may be explained by differences in pet ownership levels in these groups. For example, single people appear to be more inclined to adopt dogs [38] and have a special emotional link with their dog [18]. The same appears to be true for couples without children, who may be specially bonded with their dogs [18]. Also, in our study population dogs are importantly located in homes with children, which agrees with previous research about types of household composition preferably owning a dog (such as homes with children) [13, 37].

Participants from some areas of Spain (North East and Centre) were overrepresented. This could be due to the differences in internet use; national statistics demonstrate that North East and Central Regions exceeds the national Spanish average internet usage [39]. These are also two of the three Spanish regions with the highest populations of officially registered dogs, accounting for 29.25% of total registered Spanish dog population [40]. These regional over-representations may also reflect cultural differences in the perception of dogs, with some areas of Spain have more committed dog owners who are more willing to participate in an online survey about their relationship with their dogs.

With respect to the population of dogs included in this study, there was a balanced proportion of males and females. Almost 60% were purebred, which is similar to previous studies [4, 19].

The recruitment methods and inclusion criteria for this study introduce some limitations. The population was self-selected, and perhaps only really concerned and attached owners would be willing to participate in this kind of research project. Being recruited solely through veterinary clinics, the socioeconomic status of the participating dog owners might be skewed, as has been found in previous studies [41]; it is likely that owners who regularly attend veterinary clinics would also be those with a higher socioeconomic status, especially in the current economic climate.

### Cantril’s self-anchoring scale results

At 7.03, the mean score for Cantril’s self-anchoring scale for the study population was 11.23% higher than the reported Spanish average (6.32) [42]. Perhaps people who choose to own dogs experience a higher level of satisfaction with their lives than the general population, or maybe owning a dog makes a difference when considering one’s overall quality of life, as some studies have demonstrated that pet ownership is correlated with better health quality of life [43, 44] and psychological benefits [5]. Higher scores on Cantril’s scale have been found to be associated more strongly with income than emotional well-being [23], and positive life evaluation has been linked with educational level [45]. So, perhaps people who decide to, or can afford to, own a dog might be those who are already more materially satisfied with their lives. This is supported by some studies that show a relationship between socio-economic status and pet ownership, even though this relationship is not always direct and is dependant on the geographic and cultural context [13, 14, 46]. However, this difference may simply be the effect of self-selection of the study population. People who are more positive and satisfied with their lives might be more motivated to participate; previous research has showed that hedonic and affective factors influence people’s willingness to participate in a survey [47]. Also, as we mentioned before, some studies have shown that people who are more educated and affluent are more likely to participate in surveys [30] and high levels of satisfaction with life are linked to high income [23].

Pet ownership can have a positive or negative impact on an owner’s quality of life [48], and further research on the link between pet ownership and life satisfaction would be of value.

### Dog-owner relationship: MDORS results

Our approach to assessment of the type of dog-owner relationship was to use an existing scale that was developed using the social exchange theory [8]. This theory considers any relationship outcome as a balance between costs and benefits provided to an individual through that relationship. The MDORS [4] incorporates the assessment of both the costs and benefits of the owner-dog relationship. This contrasts with previous research that has focused exclusively either on costs or benefits [5,11].

No meaning can be ascribed to specific values of MDORS, or its subscales, due to a lack of normative data for the scale. So, it is not possible to classify owner-dog relationships as “good” or “bad” based on MDORS scores alone [4]. Within our study population, PCA-HCA identified two groups with differing relationship quality, with Group I membership being associated with generally higher values across all MDORS items. This kind of modelling and classification into different groups of quality of dog ownership has not been presented in previous research [4, 16–20]. Hence, the first and main finding of this study is that, even though we used a convenience sample of committed owners, we were able to distinguish and to characterise differing patterns of dog ownership. So, future research could apply this methodology to other different kind of samples to identify other types of dog ownership patterns.

In the O-PLS-DA model that was used to compare the two groups we identified, the variables with the strongest loadings for Group I included “I would like to have my dog near me all the time”, “My dog helps me get through tough times”, “My dog gives me a reason to get up in the morning”, and “My dog is there whenever I need to be comforted” (Fig 3). In Group I dyads, which accounted for more than half of participants, the dog was therefore endowed with many of the characteristics of a friend, partner or confidant, and provided valuable social support for the owner. This indicates that membership of Group I could be strongly influenced by owner perception of, and need for, social and emotional support from the dog. In contrast, respondents in Group II either did not experience, or did not need, this type or level of emotional support from their dog. Group I members also underrated perceived costs compared with Group II. However, it is important to remember that Group II does not represent a collection of owner-dog dyads with a poor relationship. Although Group II dog owners appear to be less emotionally dependent on their dogs, this shouldn’t be considered to be negative.. At the very least, Group II respondents are all committed dog owners who attend a veterinary clinic and were sufficiently motivated to complete the questionnaire. So, considering the previous analysis of the differences of the loadings of all MDORS variables between Group I and Group II, one may interpret that, even though all dog owners in the study are committed dog owners, Group I may represent owners with higher need for an emotionally supportive bond with their dogs and Group II may represent dog owners who are without such emotional dependence on their dogs.

This difference may relate to other aspects of the owner’s life, lifestyle and personality. To understand the factors involved in groupings of our study population, we used Binary Logistic Regression, and found four variables that were significantly associated with group membership (Table 5). Male respondents were 32.36 times more likely to be in Group 1 (the high relationship quality group), and people with a university level of maximum educational attainment were 8.65 times more likely to be in that group. For every 1-point increase in Cantril’s Self Anchoring Scale score, respondents were 23.92% (1/0.807 = 1.24 times) less likely to be in Group 1, and people with a maximum education attainment level of high school were 19.23 (1/0.052) times less likely to be in that group.

Our results therefore indicate that owner-related factors are apparently influential in the quality of the dog-owner relationship. This agrees with previous research, in which human factors have been found to have an important effect in dog-owner relationship, experiences and management, such as relinquishment [7], level of attachment to the dog [26, 49], responsible dog ownership practices [19, 26] or level of relationship [18,26]. Ultimately this may indicate that certain aspects of the owner-dog relationship may be a marker of the person’s psychological state and/or the availability of, and individual ability to use, other sources of social support.

The effect of male owners could be explained by a bias in recruitment. As previously mentioned, women constitute the majority of participants in any voluntary-recruitment study of pet ownership [4]. So, perhaps only men with a very specific, emotionally dependent, bond with their dogs were sufficiently motivated to participate in this study. This could also relate to a lack of other sources of emotional support for these individuals. As previous research has found, it seems men and women have distinct expectations for dog ownership and they differ in what the ideal characteristics of a dog are. Women seem to prefer calm and compliant dogs, while men prefer energetic, faithful and/or protective dogs [28, 29]. Men and women seem to differ also in the way they assess their dogs’ behaviour, as men tend to report more disobedience in their dogs than women [17]. Sex differences are also observed in the way people interact with their dogs; women communicate more verbally with their dogs than men [32–34]. These sex differences in communication and dog-characteristic preferences could form the basis for differences in owner-dog relationship profile. However, further research would be needed to address the effect of owner-gender on the pattern and style of dog ownership.

Maximum level of educational attainment appears influential in the quality of the owner-dog relationship, with university educated individuals being significantly more likely to have a strongly emotional relationship with their dogs, particularly when compared with people who had a high school level of maximum educational attainment. This agrees with other positive effects of higher educational level, such as better outcomes in certain tasks by the children of parents with college degrees compared to those without college degrees [50], or the positive effect of high parental socio-economic status in parent-child quality of relationship [51]. Again, this parent-child pattern could be transferred to the owner-dog pattern [32–34]. This could mean that socio-economic status, which includes dog owner’s educational level, may have an important effect on the quality of dog-owner relationship. It is also possible that educational attainment may affect expectations of pet ownership, with lower educational level leading to a greater mismatch between expectations and the reality of dog ownership. This hypothesis is supported by a previous study [28] that found a relationship between level of owner education and the characteristics of the imaginary ideal dog. The authors found a negative correlation between educational level and the expectation that the ideal dog should be sociably acceptable, energetic, faithful and protective; the more educated the owner, the less they expected from their dog. However, another explanation for the association between educational level and membership of Group 1 could be that adults with higher socioeconomic status were more likely to come from similarly high socioeconomic status families that had the resources to afford dog ownership; people with this background were more likely to have lived in dog-owning households that not only provided greater positive exposure to dogs but also enabled those children to model adult care-giving toward a pet dog. This interpretation is supported by a study showing that childhood exposure to pets positively influences adult attitudes towards pet owning [37] However, other studies have found no connection between occupation and social class, and dog ownership. A study in Central Italy showed no difference in educational level between dog owners and the non-owners [52]. Other studies showed that some social classes are more likely to own a pet-dog, like small farmers in Ireland [13]. A study in the UK showed an inverse relationship between educational level and the presence of a dog in the household [14]. Therefore, the relationship between dog ownership and socioeconomic status seems to be influenced by cultural and national factors. No studies of this kind have been carried out in Spain, so it is very difficult to place our findings in a cultural context.

Although none of the characteristics of dogs were significantly associated with Group I membership, the “mini” size of dog bordered on significance and had an odds ratio worthy of note (OR = 2.892, p = 0.07). This is consistent with results from previous research in which size of dog has been found to be related to the owner’s perception of the dog’s behavior and with the level of shared activities with the dog [17]. In that research, an inverse relationship was found between size and owner interaction; the smaller the dog, the more the owner would interact with it. The “mini dog size” factor may also be explained by the “canine cuteness effect” [53], which is a tendency for dog owners to report stronger relationships with dogs they perceive to be cute. It could be that smaller dogs could be perceived cuter than others. The number of “mini size” dogs in our sample was quite small (less than 3% of the sample population), which might explain the borderline significance.

We also found that for every one-point increase in general satisfaction with life (Cantril’s Self Anchoring Scale), the person was 1.24 times less likely to be in Group I. Cantril’s Scale is a measure of life evaluation, which is much more closely related to income and education than emotional wellbeing [23]. Given that in our study higher educational level was associated with a more emotionally dependent relationship, it might be that the effect of increasing Cantril scores was predominantly related to income and wealth for our study participants. Those with higher Cantril scores might have been more materialistic in outlook or it might be that material wealth, and in particular income, enabled individuals to engage in activities that reduced the value of social support from the dog (such as being able to spend more time with family and friends). Alternatively, perhaps the less a person is satisfied with their life, the more they look for social support [45, 54]. Dogs are considered an element of people’s social network [55] and a buffer of negative impacts [56]; perhaps those people who are less satisfied with their life situation look for support and comfort from their dog companion. However, in a US study, for the income range to $75,000 emotional wellbeing also rises with annual income [23], with the median income in the USA being $51,190 at that time [57]. If, as is likely, participants in our study were mostly middle-income earners, and for this population Cantril scores were an indirect measure of income then it is possible that increased Cantril scores were indicative of generally increased emotional wellbeing, which would reduce the value of social support available from the dog.

## Conclusions

By using a scale that applies the social exchange theory to measure costs and benefits of the owner-dog relationship [8], we have been able to identify groups with systematically different patterns of dog-owner relationship. This difference related mostly to the benefits of the owner-dog relationship (emotional closeness and level of interaction). This approach could represent a useful method for the detection of different owner-dog relationship patterns in other populations. Moreover, future studies could investigate the implications of those different owner-dog relationship patterns and consider measures to prevent their possible negative implications.

This study found an association between the quality of dog-ownership pattern and a number of owner characteristics; the owner’s sex and maximum level of educational attainment appeared to be the most important factors. These results may have implications for population selection and demographic profiling in studies that incorporate assessments of the owner-dog bond. However, more research is needed to identify dog characteristics and precise profiles related to different dog-ownership patterns.

Our results also suggest a somewhat surprising relationship between the type of owner-dog relationship and the owner’s evaluation of life satisfaction. The complexity of interpreting this finding suggests that it should be regarded as a priority for further human-animal bond research.

Findings from this study could be used to advance cross-cultural validation of the MDORS scale, as other researchers have done in other non-Anglo-Saxon cultures [16].

Since our study found differences in owner-dog relationship in a self-selected population of owners recruited through veterinary clinics; further research could use the same methods to explore dog-ownership patterns in non-committed or less responsible dog owners. This might help to identify factors of risk for unsuccessful and less responsible dog ownership and its negative consequences.

## Supporting Information

S1 File Complete collected data of our study sample. (XLS)

S2 File Original Ethics Approval Certificate from the Clinical Research Ethics Committee of the Parc de Salut Mar Medical Research Institute. (PDF)

S3 File English translation of the Ethics Approval Certificate from the Clinical Research Ethics Committee of the Parc de Salut Mar Medical Research Institute. (PDF)
