# Supplementary material for: Highly Educated Men Establish Strong Emotional Links with Their Dogs: A Study with Monash Dog Owner Relationship Scale (MDORS) in Committed Spanish Dog Owners
Source: PLoS One. 2016 Dec 29;11(12):e0168748. doi: 10.1371/journal.pone.0168748 (PMC5199054; doi:10.1371/journal.pone.0168748)
Supplement: S2 File — (PDF) [file pone.0168748.s002.pdf]

Parc de Salut Barcelona MAR

Informe del Comité Ético de Investigación Clínica

Doña Mª Teresa Navarra Alcrudo Secretaria del Comité Ético de Investigación Clínica del Parc de Salut Mar

CERTIFICA

Que éste Comité ha evaluado el proyecto de investigación clínica nº 2016/6864/I titulado *"Highly Educated Men Establish Strong Emotional Links with Their Dogs: a Study with MDORS Scale in Committed Spanish Owners"*, propuesto por la Dra. PAULA CALVO SOLER, del Grupo de Investigación en Angustia, trastornos afectivos y esquizofrènia del Instituto Hospital del Mar de Investigacions Mèdiques-IMIM

Y que considera que:

Se cumplen los requisitos necesarios de idoneidad del protocolo en relación con los objetivos del estudio y están justificados los riesgos y molestias previsibles para el sujeto.

La capacidad del investigador y los medios disponibles son apropiados para llevar a cabo el estudio.

El alcance de las compensaciones económicas que se solicitan están plenamente justificadas.

Y que éste Comité acepta que dicho proyecto de investigación sea realizado en el IMIM por la Dra. PAULA CALVO SOLER, como investigadora principal tal como recoge el ACTA de la reunión del día 7 de Junio de 2016

Lo que firmo en Barcelona, a 14 de Junio de 2016

COMITÈ ÈTIC D'INVESTIGACIÓ CLÍNICA
CEIC - PARC DE SALUT MAR

Firmado: ....
Doña Mª Teresa Navarra Alcrudo

CONSORCI MAR PARC DE SALUT DE BARCELONA I NIF: S-0800471E PAPER ECOLÒGIC

CEIC – Parc de Salut MAR
Dr. Aiguader, 88 I 08003 Barcelona I Telèfon 93 316 06 77 I Fax 93 316 06 36
ceic-psmar@imim.es | www.parcdesalutmar.cat
