# Supplementary material for: Highly Educated Men Establish Strong Emotional Links with Their Dogs: A Study with Monash Dog Owner Relationship Scale (MDORS) in Committed Spanish Dog Owners
Source: PLoS One. 2016 Dec 29;11(12):e0168748. doi: 10.1371/journal.pone.0168748 (PMC5199054; doi:10.1371/journal.pone.0168748)
Supplement: S3 File — (PDF) [file pone.0168748.s003.pdf]

REPORT FROM THE CLINICAL RESEARCH ETHICS COMMITTEE

Ms Mª Teresa Navarra Alcrudo Secretary of the Clinical Research Ethics Committee of the Parc de Salut Mar.

CERTIFIES

That this committee has assessed the clinical research project number 2016/6864/I entitled "*Highly Educated Men Stablish Strong Emotional Links with Their Dogs: a Study with MDORS Scale in Committed Spanish Owners*", proposed by PAULA CALVO SOLER, from the Group of research in anxiety, affective disorders and schizophrenia of the Hospital del Mar Medical Research Institute- IMIM.

And considers that:

The suitability requirements are met in relation to the objectives of the study and the predictable risks and inconveniences for the subjects are justified.

The researcher abilities and the available resources are appropriate to conduct the study.

The extent of the requested economic compensations is completely justified.

And that this Committe accepts that the above mentioned research project is conducted in IMIM by PAULA CALVO, as the principal researcher as it is recorded in the minutes of the meeting of the 7th June 2016.

Signed by Mª Teresa Navarra Alcrudo in Barcelona, 14th June 2016.
